# Proteomic Dynamics of *Taxus chinensis* var. *mairei* Seeds During Natural Stratification

**DOI:** 10.3390/life16101703

**Published:** 2026-10-11

**Authors:** Dandan Ma, Jia Yang, Yanting Zhang, Jiacheng Tong, Songzhe Li, Jiayi Zhang, Yusen Hong, Fangzhou Zhu, Ziying Wang, Man Zhou, Yan Xu, Shunbao Lu, Yanjie Zhang

**Affiliations:** College of Life Sciences, Jiangxi Normal University, Ziyang Rd. 99, Nanchang 330022, China

**Keywords:** *T. chinensis* var. *mairei*, stratification, differentially expressed proteins, seed

## Abstract

*Taxus chinensis* var. *mairei* is valued for paclitaxel but suffers from poor regeneration and wild resource scarcity. To elucidate seed dormancy mechanisms and support large-scale cultivation, seeds were stratified under natural conditions for 9 months and subjected to physiological and proteomic analyses. During stratification, ABA increased significantly from 0 to 3 months, showed a slight non-significant decrease from 3 to 6 months, and then increased significantly to reach its highest level at 9 months; GA3 increased significantly from 0 to 3 months and remained stable thereafter. IAA rose to a peak at 6 months and then declined. Concurrently, soluble protein peaked then declined, free amino acids showed the opposite trend, and soluble sugar, starch, and fat content continuously decreased; peroxidase (POD) activity increased then decreased, while acid phosphatase (ACP) activity decreased then increased. TMT-based quantitative proteomics identified 3047 differentially expressed proteins (DEPs were defined as protein with fold change >1.2 or <0.83 and *p* < 0.05) and Benjamini–Hochberg adjusted *p* < 0.05. GO annotation indicated enrichment in metabolic and catalytic processes, with more DEPs detected in late versus early stages. KEGG pathway analysis implicated DEPs in endoplasmic reticulum protein processing, fatty acid metabolism, carbohydrate metabolism, peroxisome pathways, and hormone signal transduction, with candidate proteins including calreticulin, protein disulfide isomerase, ACCase, acyl-CoA oxidase, phosphofructokinase, glyceraldehyde-3-phosphate dehydrogenase, catalase, PYR/PYL, and SRK2; these candidates require future functional validation. Collectively, these findings provide insights into the physiological and proteomic changes during natural stratification that are associated with progressive dormancy alleviation in *T. chinensis* var. *mairei* seeds.

## 1. Introduction

Seed dormancy is an adaptive trait that prevents premature germination under unfavorable conditions, yet excessive dormancy causes delayed and uneven germination in the field [1]. Defined as the failure of viable seeds to germinate under otherwise suitable conditions [2], dormancy is governed by multiple genes [3] and modulated by genotype, reactive oxygen species, and phytohormones [4], as well as by environmental cues such as light and temperature [2]. Seasonal soil temperature fluctuations drive the annual dormancy–non-dormancy cycle, synchronizing germination with favorable seasons [5].

Under natural conditions, dormancy release is regulated by soil temperature and hormone signaling—particularly ABA, GA, and IAA—with ABA playing a central role [6,7]. Concurrently, seed storage reserves are mobilized: storage proteins, carbohydrates, and lipids are catabolized, with starch converting to soluble sugars or other small molecules to fuel embryo growth [8,9]. The decline in soluble sugars during stratification suggests they serve as the primary carbon source for embryo development [10], while in pear and oat seeds, fat may be converted to soluble sugars for the same purpose [11,12]. However, the hormonal regulation of dormancy is species-specific. For instance, rice dormancy is effectively released by high temperature and humidity, independent of ABA and GA content [13]. *Acer pseudoplatanus* seeds, which exhibit deep physiological dormancy, require 3–4 months of cold stratification for germination, a process accelerated by exogenous GA after partial stratification [14,15]. Similarly, *Cariniana legalis* seed germination is negatively correlated with temperature and storage time, remaining higher at 6 °C than at 25 °C over two years [16].

Proteomic approaches have advanced our understanding of these processes. Two-dimensional gel electrophoresis (2-DE) has revealed that differentially expressed proteins (DEPs) involved in energy metabolism, protein synthesis, redox regulation, and hormone signaling are key to dormancy release in rice, sycamore, rose, and soybean [17,18,19,20]. For example, reduced ABA signaling (via PYR/PYL, PP2C, and SnRK2) and upregulation of heat shock proteins and chaperones have been linked to germination promotion in rice and sycamore [17,18]. However, most such findings derive from laboratory-controlled experiments; proteomic studies under natural stratification—which more faithfully reflect field conditions—remain scarce, especially for deep-dormancy species such as *T*. *chinensis* var. *mairei*.

*T. chinensis* var. *mairei* is an endangered species, classified as a first-class nationally protected wild plant in China, and is valued for its paclitaxel content [21]. Its seeds exhibit deep dormancy, and understanding this trait is critical for germplasm conservation and utilization. Yet previous molecular studies on it have been largely laboratory-based, with limited long-term investigation under natural conditions [22]. While Chen et al. recently reported a multi-omics characterization of germinating *T. chinensis* var. *mairei* seeds, the proteomic changes that occur across the full course of natural stratification from the initial dormant state through progressive dormancy alleviation remain to be characterized [22]. However, most proteomic studies of seed dormancy have been conducted under laboratory-controlled conditions. Here, we buried seeds in their native soil natural stratification for nine months and periodically profiled DEP changes, aiming to characterize the proteomic dynamics of dormancy alleviation during stratification in naturally stratified seeds, thereby providing a temporal view of the proteomic shifts that accompany dormancy alleviation under field conditions.

## 2. Materials and Methods

### 2.1. Plant Materials

*T. chinensis* var. *mairei* seeds were collected in natural populations distributed in Xiushui County, Jiangxi Province (114°32′48″ E, 29°01′35″ N) in the winter of 2021. All collection procedures complied with the Regulations of China on the Protection of Wild Plants and the local regulations for first-class protected species. Fresh seeds were promptly placed into 12 independent nylon mesh bags (mesh size 0.5 mm; 10 cm × 15 cm) to retain seeds while permitting water and gas exchange with the surrounding soil, each containing seeds from the same homogenized seed lot, and randomly assigned to 4 sampling time points (0, 3, 6, and 9 months; 3 bags per time point). All bags were buried in situ at a depth of 2 cm in the native soil habitat for 9 months (4 January 2022–4 October 2022). 

At each sampling time (0, 3, 6, and 9 months), seeds were retrieved, immediately frozen in liquid nitrogen, and stored at −80 °C until hormone extraction and proteomic analysis. To characterize the temporal distribution of DEPs during seed dormancy under natural stratification, DEPs were quantified using TMT labeling coupled with LC-MS/MS. Because the site was remote and disturbance needed to be minimized, continuous monitoring of soil temperature, moisture, and other microenvironmental variables was not conducted during the 9-month field experiment.

### 2.2. Determination of Plant Hormones

An enzyme linked immunosorbent assay (ELISA, Shanghai Enzyme-linked Biotechnology Co., Ltd., Shanghai, China) was used to determine the content of ABA, GA_3_ and IAA from 0.3 g seeds [23].

Samples were extracted with 10 mL 80% (*V*/*V*) methanol containing 1 mmol/L Butylated hydroxytoluene for 12 h in the dark at 4 °C and used the kits of ABA, GA_3_ and IAA to determine the levels of plant hormones in the samples by competitive inhibition ELISA. The microporous plates were coated with purified antibodies of plant hormones (ABA, GA_3_, IAA) to prepare solid phase antibodies. The plant hormones were successively added to the microporous plates, which were coated with monoclonal antibodies, and then conjugated with horseradish peroxidase-labeled plant hormone antibodies to form antibody–antigen–enzyme-conjugated antibody complexes. After thorough washing, the substrate TMB (3,3′,5,5′-Tetramethylbenzidine) was added for chromogenic reaction.

Before the experiment, the reagents and samples were added to the coated wells and reacted for 30 min at 37 °C. The plates were washed 5 times and enzyme standard reagents were added. After reacting for 30 min at 37 °C, the plates were washed 5 times, color solution was added, and they were reacted for an additional 10 min at 37 °C. Finally, the stop solution was added to terminate the reaction. The absorbance (OD value) was measured with a microplate reader at 450 nm to build a standard curve. We then calculated the enzyme immunoassay data from the standard curve [24].

### 2.3. Determination of Storage Materials

An amount of 0.3 g of embryos was taken from each seed sample. The soluble protein content of seeds was determined by Coomassie brilliant blue method [25], soluble sugar content by the Athrone colorimetry method [26], starch content by the Iodine colorimetric method [27], fat content by the Soxhlet extraction method [28] and amino acids by the Ninhydrin colorimetry method [29].

### 2.4. POD Activity and ACP Determination

Exactly 0.3 g of embryos were ground into homogenate with 2 mL phosphate buffer on ice, then washed with 6 mL phosphate buffer and centrifuged at 4000 r/min for 20 min at 4 °C, and the supernatant was kept. The reaction mixture consisted of 50 mL phosphoric buffer, 28 μL guaiacol and 19 μL hydrogen peroxide (30%), which were mixed evenly and stored at 4 °C. A total of 3 mL of reaction mixture was mixed with 20 μL enzyme extract and was shaken evenly. In the control experiment, 20 μL phosphoric buffer was used to replace the enzyme extract. According to the guaiacol method to measure the activity of POD, the activity of the guaiacol oxidation reaction per minute was tested by a spectrophotometer at a wavelength of 470 nm at 20 °C [30]. 

The activity of ACP was determined by the reaction of the sample with p-nitrophenyl phosphate disodium salt, which produced the amount of p-nitrophenol. Amounts of 1.0 mL of 0.1 mol/L NaOH, 1 mL acetate buffer (pH = 7.5), 0.1 mL of enzyme extract from the sample and 0.1 mL of 0.018 mol/L p-nitrophenyl phosphate disodium salt were mixed and placed in a 30 °C incubator to react for 10 min. Then 1.0 mL of 0.5 mol/L NaOH was added to stop the reaction and the absorbance value was measured at 400 nm. One unit of ACP activity was defined as the amount of enzyme that releases 1 nmol of p-nitrophenol per minute per milligram of protein under the assay conditions [31]. 

### 2.5. Protein Extraction

The protein extraction of samples was carried out using the tandem mass tag (TMT) quantitation protocol with modifications [26]. Frozen hulled kernel samples were transferred to a shock tube and supplemented with an extraction solution containing 1% (*w*/*v*) polyvinylpolypyrrolidone (PVPP) and 5 mM benzyltriphenylphosphonium chloride (BPP) in 50 mM Tris-HCl (pH 7.5). After grinding, the samples were centrifuged and the supernatant was mixed with Tris-phenol. The final protein pellet was dissolved in lysis buffer consisting of 50 mM Tris-HCl (pH 7.5), 2% (*w*/*v*) SDS, 10 mM DTT, 1 mM PMSF, and 1% (*v*/*v*) protease inhibitor cocktail. The mixtures were subjected to three cycles of oscillation using a high-throughput tissue grinder, each lasting 40 s. After grinding, the samples were centrifuged at 12,000 r/min for 20 min at 4 °C. The supernatant was collected and mixed with an equal volume of Tris-phenol, followed by oscillation at 4 °C for 10 min. The phenolic phase was recovered after another centrifugation step (12,000 r/min, 20 min), where the solution was combined with an equal volume of PVPP, and shaken for 10 min. Finally, a five-fold volume of cold ammonium acetate-methanol buffer was added to the phenolic supernatant mixture, and proteins were precipitated at −20 °C for 8 h. After 8 h, the precipitate was collected by centrifugation at 12,000 r/min for 20 min at 4 °C.

After 8 h, the precipitate was collected by centrifugation at 12,000 r/min for 20 min at 4 °C. The pellet was then washed twice with 90% pre-cooled acetone and re-collected each time. Subsequently, the precipitate was dissolved in lysis buffer and sonicated on ice using a Scientz-IID ultrasonic homogenizer (Ningbo Scientz Biotechnology Co., Ltd., Ningbo, China) (20% power, 3 mm probe, 2 s on/3 s off, total 2 min). The protein supernatant was obtained after another centrifugation step under the same conditions (12,000 r/min, 20 min, 4 °C). Finally, the protein concentration was determined using a BCA assay kit (Thermo Fisher Scientific; Waltham, MA, USA), and the samples were stored at −80 °C.

### 2.6. Protein Digestion and TMT Labeling

The protein samples of 100 μg were taken for trypsin digestion. The protein samples reacted for 1 h at 37 °C with 100 mM TEAB (Triethylammonium bicarbonate buffer) and 100 mM TCEP (Tris (2-carboxyethyl) phosphine). Exactly 40 mM IAM (Iodoacetamide) was added and reacted for 40 min at room temperature in darkness. Then precooled acetone was added and precipitated for 4 h at −20 °C, then we collected the precipitation after centrifugation at 10,000 r/min for 20 min at 4 °C. Finally, the precipitation was dissolved in 100 μL of 100 mM TEAB and trypsin was added at a 1:50 trypsin-to-protein mass ratio for digestion overnight at 37 °C.

The acetonitrile was added to tandem mass tags (TMTs) reagent and centrifuged by swirling, then the digested peptides were added, in which 100 μg of peptides were labeled by one unit of TMT reagent and incubated for 2 h at room temperature. Next, hydroxylamine was added to the peptide mixtures and incubated for 30 min at room temperature then blended. Finally, the mixtures were dried with vacuum concentrator. Four samples were labeled by TMTs and each sample with three biological replicates.

### 2.7. HPLC for Fractionation and Subsequent Analysis by LC-MS/MS

The TMT-labeled samples were dissolved in 2% acetonitrile (pH 10 adjusted with ammonia water) and fractionated by high-pH reverse-phase high-performance liquid chromatography (HPLC) on a Shimadzu UFLC Prominence System equipped with a C18 column (1.7 μm particles, 2.1 mm ID, 250 mm length). The flow rate was 0.3 mL/min, and the column temperature was 40 °C. Twenty fractions were collected and combined into 10 fractions by concatenation based on UV absorbance at 214 nm. The fractions were concentrated by vacuum centrifugation and dissolved in mass spectrometry buffer (2% acetonitrile and 0.1% formic acid) for the second-dimension analysis.

Liquid chromatography-tandem mass spectrometry (LC-MS/MS) detection was carried out as in a previous report [26]. The peptides were separated by the reversed-phase analytical column C18 (75 μm × 25 cm, Thermo, Waltham, MA, USA). The solvent B gradient (0.1% formic acid in 80% acetonitrile) was set as follows: 0–51 min, 8–23% B; 51–71 min, 23–29% B; 71–87 min, 29–38% B; 87–90 min, 38–48% B; 90–93 min, 48–100% B; 93–120 min, 100% B. The fluent flow rate of 300 nL/min was set in the system of EASY-nLC 1200 UPLC. The separated peptides were analyzed through Orbitrap Exploris 480 mass spectrometer (Thermo, USA). The 10 most abundant precursor ions from the survey scan (300–1600 *m*/*z*) were selected for higher-energy collisional dissociation (HCD) fragmentation at a normalized collision energy of 32. The resulting fragments were analyzed with the Orbitrap at a resolution of 35,000 with an AGC target of 2 × 10^5^, maximum injection time of 80 ms, and dynamic exclusion duration of 30.0 s. 

### 2.8. Database Search and Bioinformatics Analysis

The raw MS/MS data were processed using ProteomeDiscoverer^TM^ Software 2.4. The raw files were searched against the established database, allowing a maximum of two missed tryptic cleavages. Precursor and fragment mass tolerances were set to 20 ppm and 0.02 Da, respectively. Peptide-spectrum matches were filtered at a false discovery rate (FDR) ≤ 0.01. For protein-level differential expression, the BH procedure was applied to control the false discovery rate. Proteins were considered differentially expressed when the BH-adjusted *p*-value was <0.05 and the fold change was >1.2 or <0.83. A more stringent threshold of *p* < 0.01 was also tested as a sensitivity analysis. For protein-level differential expression, a nominal *p* < 0.05 was initially calculated, and multiple-testing correction was performed using the Benjamini–Hochberg procedure. 

Proteins were considered differentially expressed only if they had a fold change >1.2 or <0.83 and a BH-adjusted *p*-value < 0.05. Three comparison groups (0 months/3 months, 3 months/6 months, and 6 months/9 months) were constructed, with three biological replicates per stage. Proteins were defined as up-regulated when fold change >1.2 and q < 0.05, and down-regulated when fold change <0.83 and *p* < 0.05. Three comparison groups (0 months/3 months, 3 months/6 months and 6 months/9 months) were constructed, with three biological replicates for per stage. Proteins were defined as up-regulation when the fold change was >1.2 and down-regulation when the fold change was <0.83. The DEPs of GO function and KEGG pathway annotation were analyzed by the Meiji Bioinformatics cloud platform (https://vip.majorbio.com).

### 2.9. Statistical Analysis

The physiological and biochemical parameters for all treatments were analyzed using one-way ANOVA in SPSS 27.0, followed by least significant difference (LSD) tests at a significance level of *p* < 0.05 after BH-FDR correction for multiple comparisons. For hormone quantification, three technical replicates per sample were averaged before statistical analysis. Data are presented as mean ± standard error (SE).

## 3. Results

### 3.1. Changes in Endogenous Hormones During Natural Stratification

**Figure 1:**
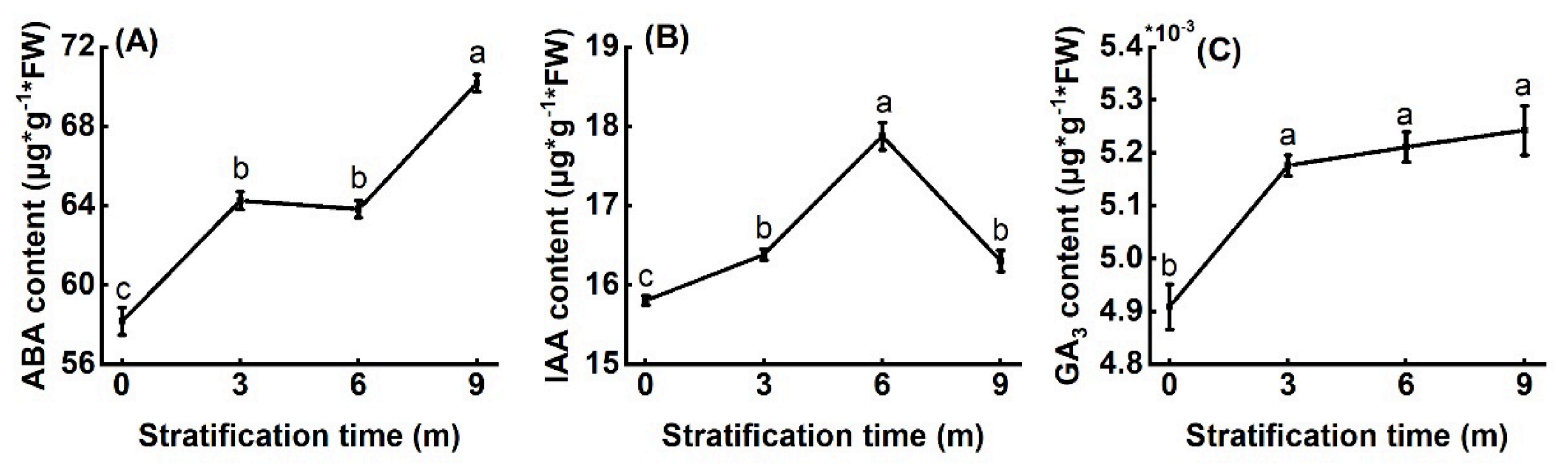
Changes in ABA (**A**), IAA (**B**), GA_3_ (**C**) during natural stratification for 9 months. Different lowercase letters above the bars indicate significant differences among stratification time points (one-way ANOVA, LSD test, *p* < 0.05). Data are presented as mean ± SE (n = 3).

There was a significant increase in ABA and GA_3_ content of the seeds during the natural stratification after 9 months (Figure 1A–C), whereas IAA peaked at mid-stratification and then declined. The content of ABA increased significantly from 0 to 3 months (*p* < 0.05), decreased slightly from 3 to 6 months, and increased significantly from 6 to 9 months (*p* < 0.05), reaching its highest value at 9 months (70.18 μg/g FW). The content of IAA increased significantly from 0 to 6 months (*p* < 0.05) and peaked at 6 months (17.88 μg/g FW), and then decreased significantly. The content of GA_3_ increased significantly from 0 to 3 months (*p* < 0.05), and then showed no significant change from 3 to 9 months.

### 3.2. Changes in Activities of POD and ACP During Natural Stratification

**Figure 2:**
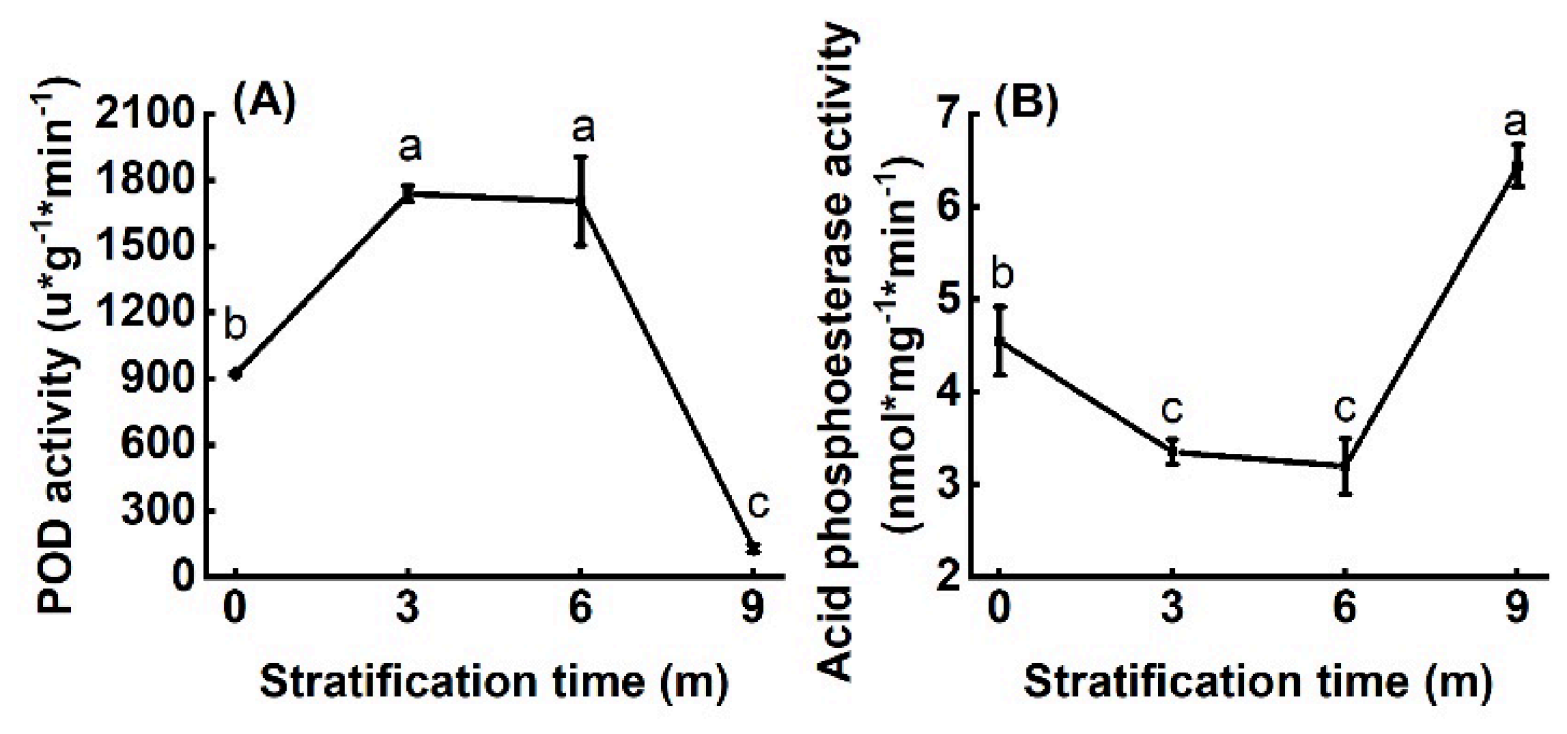
Changes in activities of POD (**A**), ACP (**B**) during natural stratification for 9 months. Different lowercase letters above the bars indicate significant differences among stratification time points (one-way ANOVA, LSD test, *p* < 0.05). Data are presented as mean ± SE (n = 3).

POD activity increased significantly from 0 to 3 months, with the highest value (1739.13 μ/g/min) at 3 months, slightly decreasing at 6 months, and decreasing significantly at 9 months (Figure 2A). ACP activity decreased significantly from 0 to 3 months, slightly decreased from 6 months, and increased significantly at 9 months (Figure 2B).

### 3.3. Seed Nutrient Metabolism During Natural Stratification

**Figure 3:**
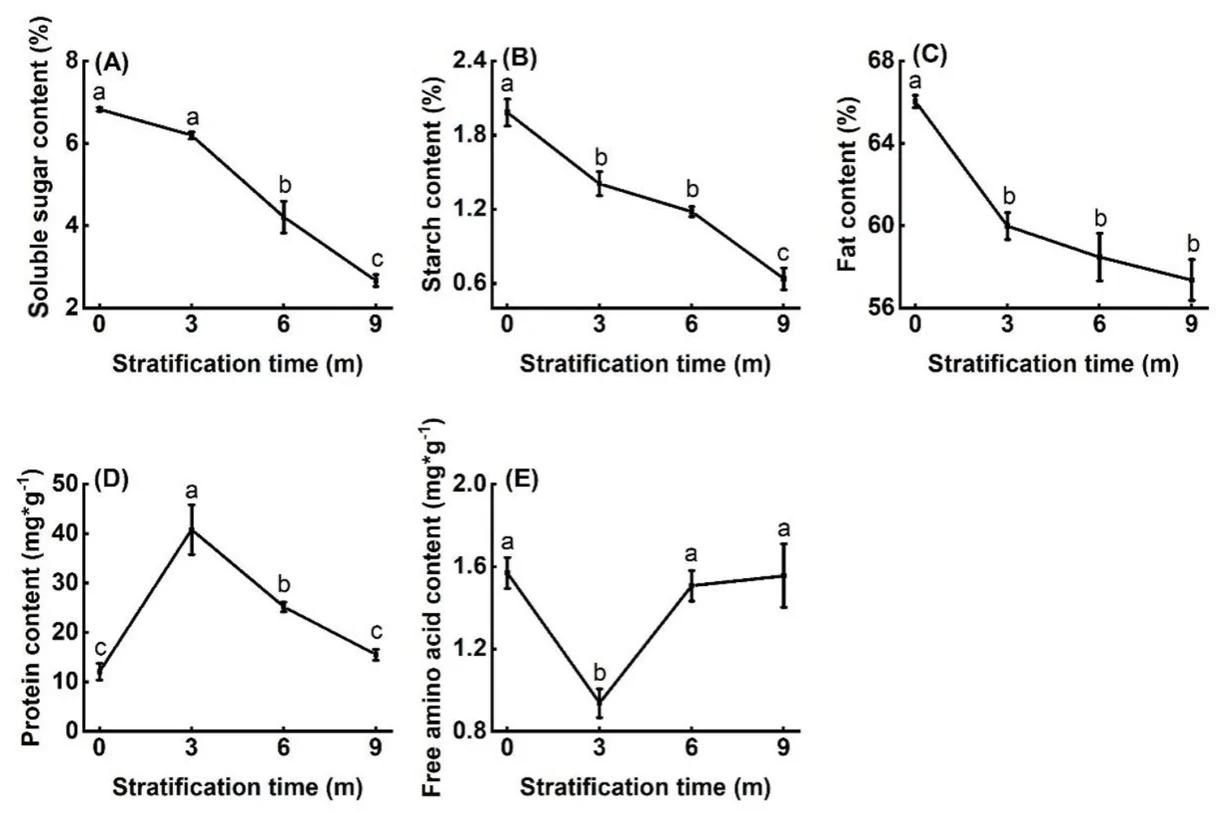
Changes in the nutrient content of soluble sugar (**A**), starch (**B**), fat (**C**), protein (**D**), free amino acid (**E**) during natural stratification for 9 months. Different lowercase letters above the bars indicate significant differences among stratification time points (one-way ANOVA, LSD test, *p* < 0.05). Data are presented as mean ± SE (n = 3).

The contents of soluble sugar, starch and fat of seeds significantly decreased during natural stratification (Figure 3A–C). 

The content of soluble protein increased significantly from 0 to 3 months and peaked at 3 months, decreasing significantly afterwards (Figure 3D). However, free amino acid content decreased significantly from 0 to 3 months, increased significantly at 6 months, and slightly increased at 9 months (Figure 3E). The soluble protein and free amino acid content varied in the ranges of 12.07–40.8 and 0.93–1.56 mg g^−1^.

### 3.4. Changes in Protein Abundance During Seed Natural Stratification

The TMT experiment generated 255,334 spectra, of which 27,622 were successfully matched, leading to the identification of 3754 protein groups. A total of 3047 proteins were differentially expressed (*p* < 0.05) during natural stratification.

**Figure 4:**
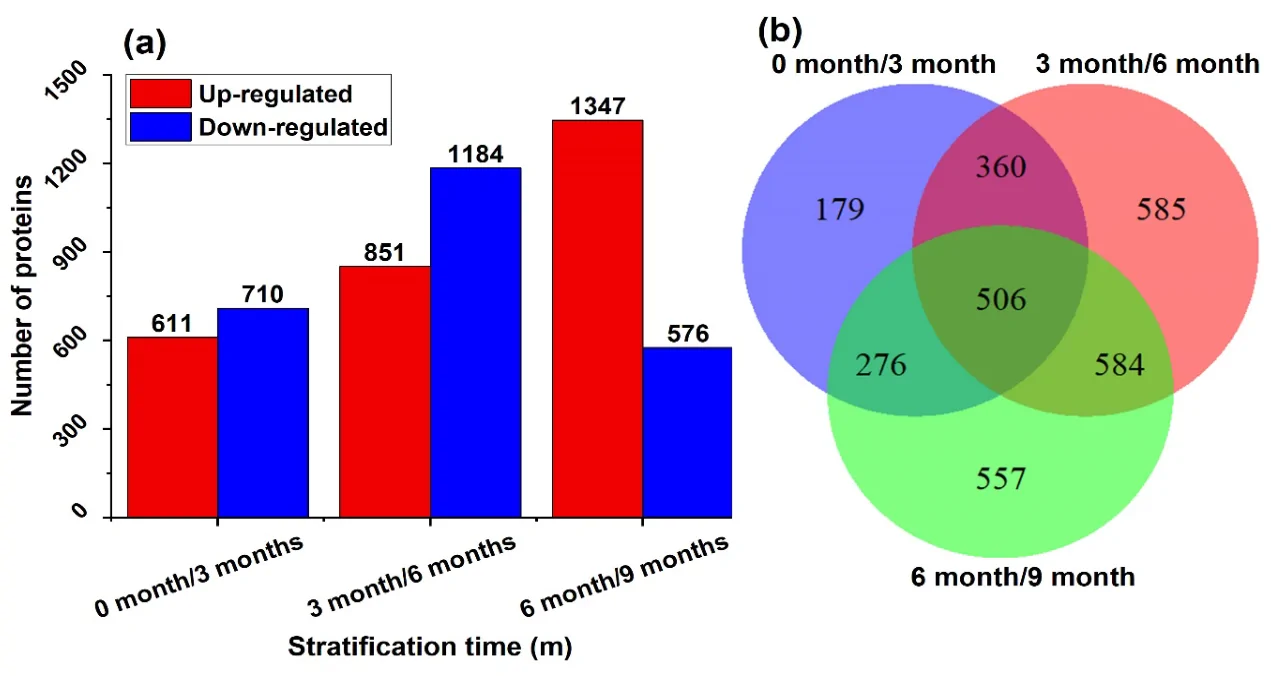
The profile of DEPs. (**a**) The number of DEPs identified in different comparisons during natural stratification. (**b**) Venn diagram showing the number of proteins identified in the three different contrast groups (0 months/3 months (blue), 3 months/6 months (red), and 6 months/9 months (green)).

Among these, 1321 DEPs were identified in the 0 months/3 months comparison, with 611 up-regulation and 710 down-regulated. The number of DEPs increased markedly in the 3 months/6 months group, reaching 2035 (851 up-regulation and 1184 down-regulated). In the 6 months/9 months, 1923 DEPs were identified, including 1347 up-regulation and 576 down-regulated (Figure 4a). In addition, 506 DEPs were common across all stratification stages, while 179, 585, and 557 were specific to the 0–3-month, 3–6-month, and 6–9-month intervals, respectively (Figure 4b).

### 3.5. GO Analyses of DEPs in Response to Seed Natural Stratification

**Figure 5:**
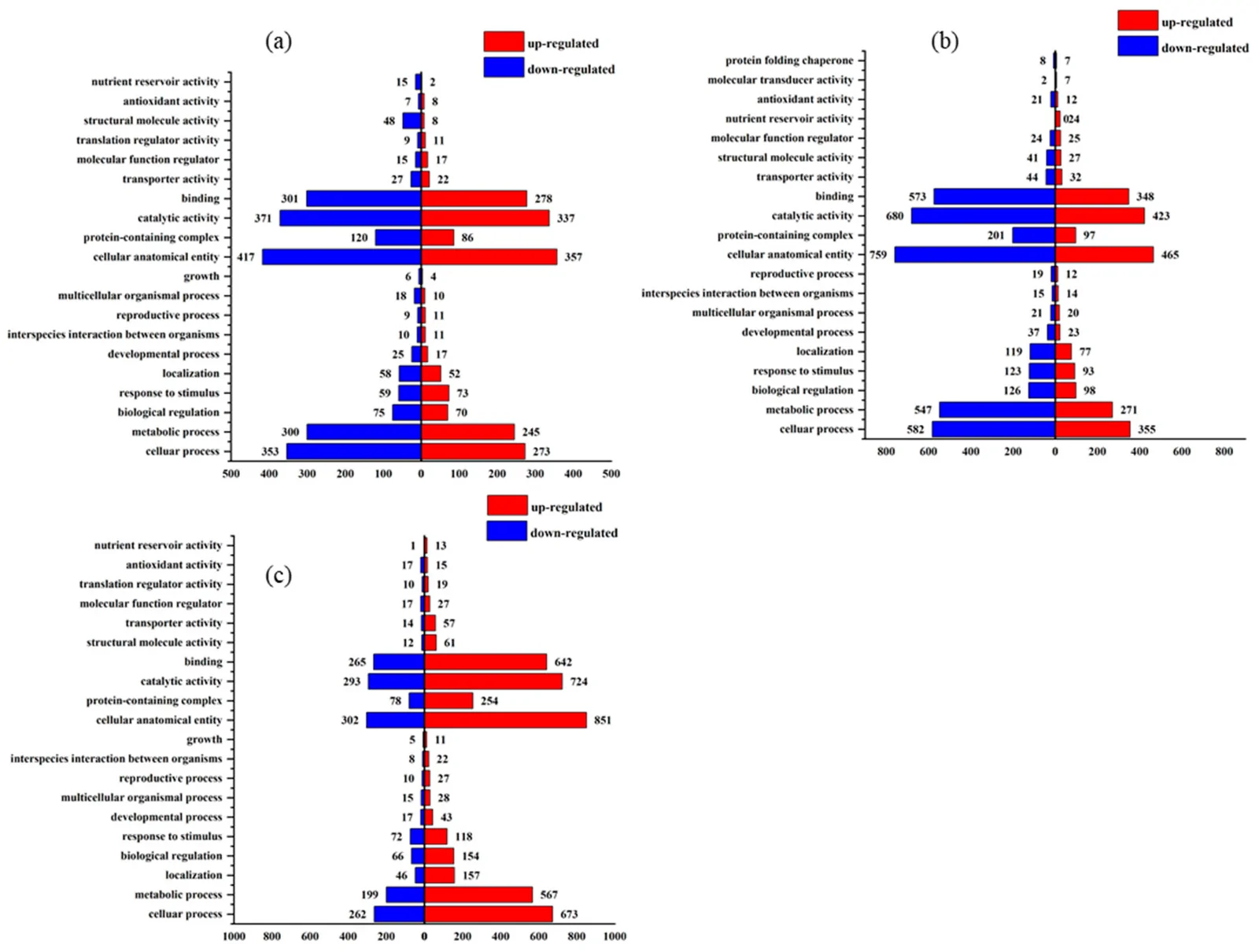
GO Secondary Classification of DEPs. The *x*-axis represents the number of enriched DEPs by GO annotation. (**a**) The DEPs of 0 months/3 months by GO; (**b**) the DEPs of 3 months/6 months by GO; (**c**) the DEPs of 6 months/9 months by GO.

A total of 3387 DEPs were annotated into the GO database. The top 20 DEPs in protein abundance were categorized into three major functional classes: biological process, cellular component, and molecular function. Among the three different stages, the most enriched biological processes included cellular process, metabolic process and biological regulation. Within cellular components, cellular anatomical entity and protein-containing complex were predominant. For molecular functions, catalytic activity and binding were highly represented among DEPs (Figure 5a–c). Notably, molecular transducer activity and protein folding chaperone were exclusively identified at 3 months/6 months (Figure 5b); whereas, growth and translation regulator activities were only observed in the 0 months/3 months and 6 months/9 months comparisons (Figure 5a,c). Comparative analysis revealed a higher number of DEPs at later stages than at earlier ones, suggesting an activation of metabolic processes during late stratification.

### 3.6. KEGG Analyses of DEPs in Response to Seed Natural Stratification

#### 3.6.1. KEGG Analyses of DEPs Which Processing in Endoplasmic Reticulum

**Figure 6:**
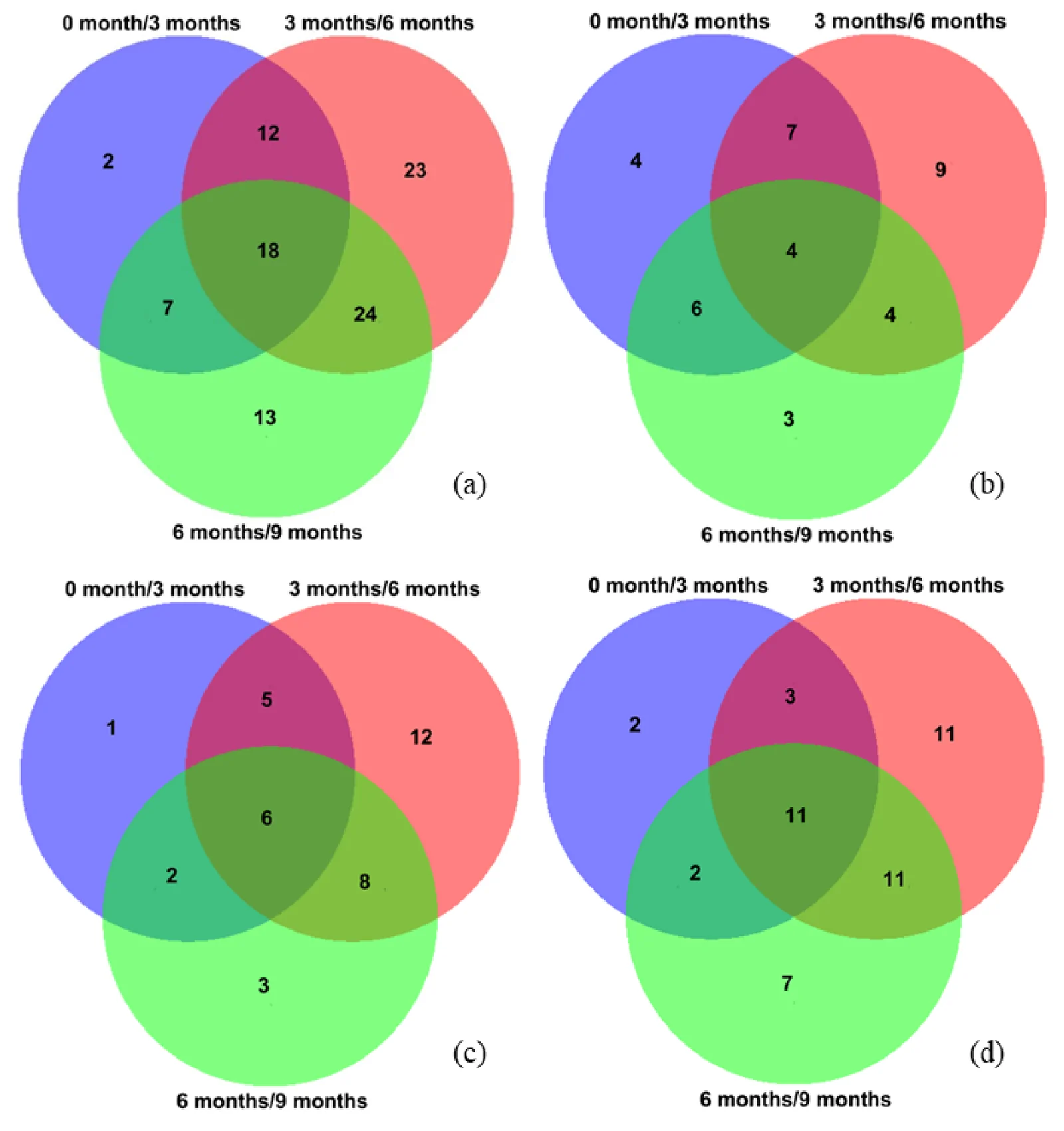
Venn diagram showing the number of proteins involved in (**a**) protein processing in the endoplasmic reticulum, (**b**) fatty acid biosynthesis, (**c**) fatty acid degradation, and (**d**) starch and sucrose metabolism. The three comparison groups are: 0 months/3 months (blue), 3 months/6 months (red), and 6 months/9 months (green).

KEGG pathway analysis showed that the majority of DEPs were primarily associated with energy metabolism (including protein, carbohydrate and fat), peroxisomal pathways, and plant hormone signal transduction. In the endoplasmic reticulum-related protein processing pathway, 39 DEPs were identified in the 0 months/3 months comparison group, 77 in the 3 months/6 months group, and 62 in the 6 months/9 months group. Furthermore, there were 18 common DEPs across all three comparisons groups, along with two, 23, and 13 specific DEPs in each respective group involved in this pathway (Figure 6a). 

#### 3.6.2. KEGG Analysis of DEPs in Fatty Acid Biosynthesis and Degradation

A total of 37 unique DEPs were associated with fatty acid biosynthesis and 37 unique DEPs with fatty acid degradation across the three comparison groups. In the 0 months/3 months comparison, 21 DEPs participated in fatty acid biosynthesis and 14 in degradation. For the 3 months/6 months comparison, the corresponding numbers were 24 and 31, respectively. For the 6 months/9 months comparison, 17 DEPs were involved in biosynthesis and 19 in degradation. Venn diagram analysis showed that four DEPs were shared among all three comparisons for fatty acid biosynthesis, with four, nine, and three unique to each comparison, respectively (Figure 6b). For fatty acid degradation, six DEPs were shared among all three comparisons, while one, 12, and three were specific to each comparison (Figure 6c). After removing redundancy, the total number of unique DEPs was 37 for both pathways. 

#### 3.6.3. KEGG Analyses of DEPs Involved in Carbohydrate Metabolism

**Figure 7:**
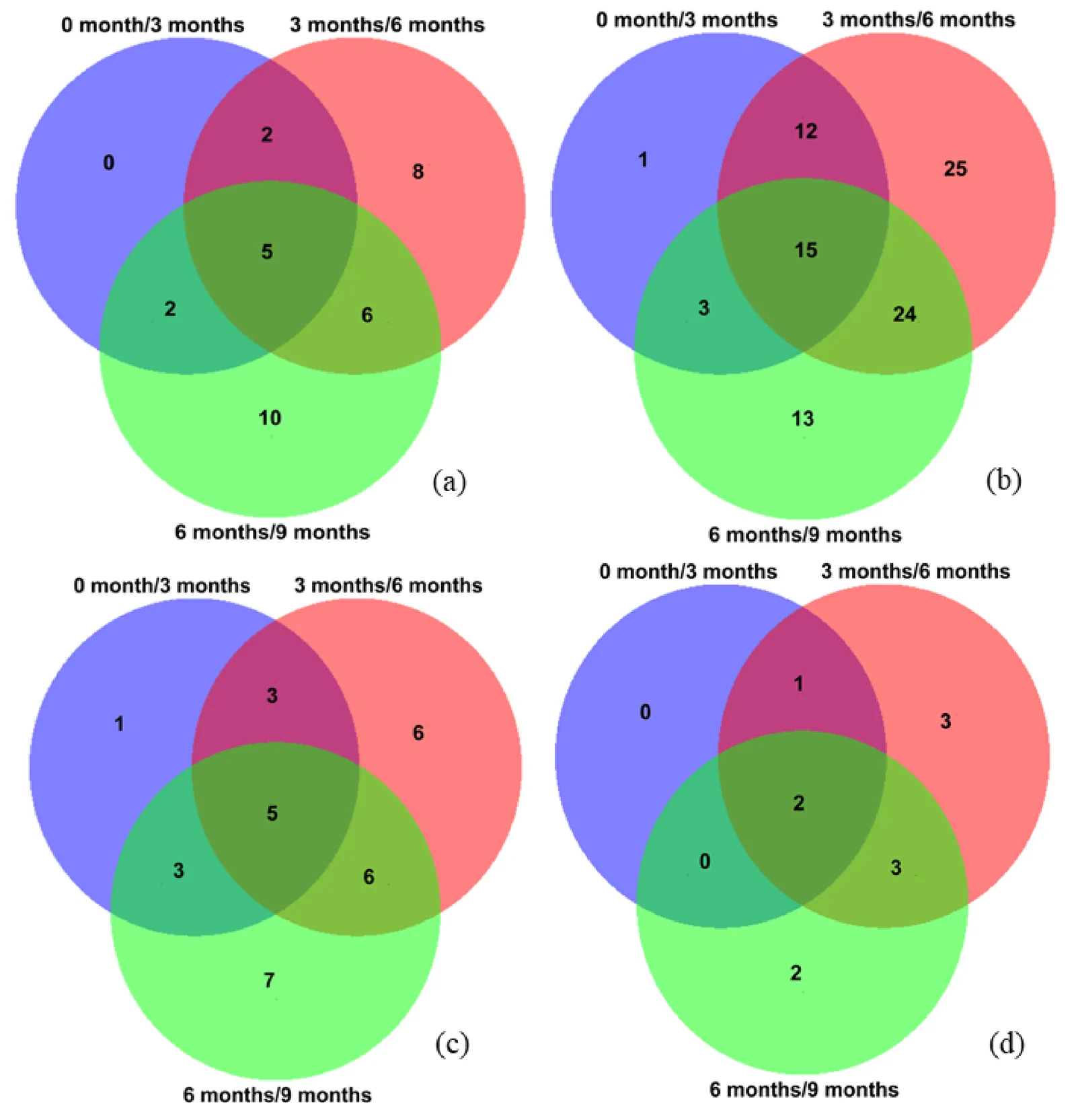
Venn diagram showing the number of proteins involved in fructose and mannose metabolism (**a**), glycolysis/gluconeogenesis (**b**), peroxisome (**c**) and plant hormone signal transduction (**d**) identified in the three different comparison groups (0 months/3 months (blue), 3 months/6 months (red), and 6 months/9 months (green).

A total of 18, 36, and 31 DEPs from the 0 months/3 months, 3 months/6 months, and 6 months/9 months comparison groups, respectively, were involved in starch and sucrose metabolism (Figure 6d). Similarly, nine, 21, and 23 DEPs from the respective comparison groups participated in fructose and mannose metabolism (Figure 7a). For glycolysis/gluconeogenesis, a total of 31, 76, and 55 DEPs were identified in the 0 months/3 months, 3 months/6 months, and 6 months/9 months comparisons, respectively, with a union of 93 unique DEPs across all three groups (Figure 7b). Among these, 15 DEPs were shared by all three comparison groups, while one, 25, and 13 DEPs were specific to the 0 month/3 months, 3 months/6 months, and 6 months/9 months comparisons, respectively. In addition, 12 DEPs were shared between the 0 months/3 months and 3 months/6 months comparisons, three between 0 months/3 months and 6 months/9 months, and 24 between 3 months/6 months and 6 months/9 months. 

#### 3.6.4. KEGG Analyses of DEPs in Response to Stress

KEGG analysis revealed that the stress-responsive DEPs were enriched in the peroxisome pathway. A total of 53 DEP identifications (non-unique across comparisons) were associated with the peroxisome pathway, corresponding to 31 unique DEPs after removing redundancy across the three comparisons (Figure 7c). Among the three comparison groups, 0 months/3 months, 3 months/6 months, and 6 months/9 months, 12, 20, and 21 DEPs were detected, respectively. These groups shared five common DEPs, while containing one, six, and seven unique DEPs, respectively (Figure 7c).

#### 3.6.5. KEGG Analyses of DEPs Involved in Plant Hormone Signal Transduction

A total of three, nine, and seven DEPs from the 0 months/3 months, 3 months/6 months, and 6 months/9 months comparison groups, respectively, were involved in plant hormone signal transduction. Among these, two DEPs were common across all three comparison groups, while 0, three, and two were specific to the 0 months/3 months, 3 months/6 months, and 6 months/9 months groups, respectively (Figure 7d). 

## 4. Discussion

### 4.1. Dynamic Changes in Storage Reserves and Their Role in Dormancy Release During Natural Stratification

Seed dormancy release is a complex physiological process that requires the breakdown of storage reserves-including proteins, fats, and starches to supply essential energy for embryo growth. The opposing trends in soluble protein and free amino acid contents during stratification (Figure 3D,E) are consistent with the known mobilization of storage proteins during dormancy alleviation, in which proteins are hydrolyzed to free amino acids that can be used for new protein synthesis or energy production [32,33,34]. However, as discussed below, these trends alone do not establish a direct reversible conversion.

### 4.2. DEPs Are Involved in Protein Biosynthesis and Metabolism

During seed natural stratification, 99 DEPs were annotated to the KEGG pathway with protein processing in the endoplasmic reticulum (ER). Calreticulin (CRT), a calcium-binding molecular chaperone, positively regulates the Ca^2+^ storage capacity within the ER [35]. As Ca^2+^ signaling plays a crucial role in seed dormancy release in other species [18], the up-regulation of CRT from 3 to 9 months is consistent with an active phase of dormancy alleviation during this period. Meanwhile, protein disulfide isomerase (PDI), which facilitates disulfide bond formation, was up-regulated from 0 to 3 months and down-regulated from 3 to 9 months, suggesting that DEPs may be associated with stage-specific effects on protein conformational stability and function [36].

The cumulative comparisons against the 0-month baseline provide complementary insights beyond the consecutive comparisons. While the consecutive comparisons revealed stage-specific transitions, the cumulative comparisons identified proteins that changed monotonically across the entire stratification period. These progressive dormancy-associated proteins were enriched in translation, protein folding, and redox regulation, consistent with a sustained requirement for protein synthesis, conformational maintenance, and oxidative balance during long-term dormancy alleviation. In contrast, proteins involved in storage reserve mobilization showed more dynamic, non-monotonic patterns, suggesting that reserve utilization is stage-dependent rather than linear.

### 4.3. DEPs Are Involved in Lipid Biosynthesis and Metabolism

Acetyl-CoA carboxylase (ACCase) catalyzes the conversion of acetyl-CoA to malonyl-CoA, the committed and rate-limiting step in fatty acid biosynthesis [37]. In this study, ACCase was down-regulated from 0 to 3 months, suggesting a reduction in fatty acid synthesis. This is consistent with the continuous decrease in total fat content observed during stratification (Figure 3C), which indicates the mobilization of storage lipids to support embryo growth [38,39]. In contrast, several DEPs involved in fatty acid β-oxidation were identified, including acyl-CoA oxidase, which catalyzes the rate-limiting step in fatty acid degradation [40]. The coordinated down-regulation of ACCase and the presence of β-oxidation-related DEPs suggest a metabolic shift from lipid biosynthesis to lipid catabolism during stratification. However, we note that the down-regulation of ACCase alone does not directly prove enhanced degradation; rather, it reflects reduced synthesis, while the degradation pathway is supported by the identification of specific β-oxidation enzymes and the depletion of fat reserves.

### 4.4. DEPs Are Involved in Carbohydrate Metabolism

Glyceraldehyde 3-phosphate dehydrogenase (GAPDH) catalyzes the oxidative phosphorylation of glyceraldehyde 3-phosphate (G3P) to 1,3-bisphosphoglycerate (1,3-BPG), coupled with the reduction in NAD^+^ to NADH, in the glycolytic pathway. The interconversion between G3P and dihydroxyacetone phosphate (DHAP) is catalyzed by triose phosphate isomerase (TPI), not GAPDH. In our dataset, GAPDH was identified as a specific differentially expressed protein at the 3–6-month stage, with up-regulation during this period, which is consistent with enhanced glycolytic activity and active carbohydrate metabolism during seed stratification [41,42]; however, direct measurement of glycolytic flux was not performed.

### 4.5. DEPs Involved in Stress Response

Based on the results and properties of POD, they are divided into three families, including catalase, POD, and ascorbate POD. Their main function is to clear intracellular reactive oxygen species (ROS) generated during various biological processes, protecting plants from damage. During stratification (0–9 months), 53 DEPs were annotated to the peroxisomes pathway, including copper/zinc superoxide dismutase, acyl-CoA oxidase, long-chain acyl-CoA synthetase, and catalase. These proteins are known to be involved in ROS metabolism in other systems; however, their specific roles in this study remain correlative [42,43].

### 4.6. Limitations and Future Perspectives

While our study provides a valuable temporal view of physiological and proteomic changes in buried *T. chinensis* var. *mairei* seeds during natural stratification, several limitations must be acknowledged. First, we did not monitor soil microenvironmental parameters (e.g., temperature, moisture), which would be essential for attributing specific physiological shifts to environmental triggers such as winter cold or spring warming. However, because seeds were buried in their native habitat, our results reflect integrative responses to natural field fluctuations, though the lack of continuous environmental data limits causal interpretation. Second, our TMT-based proteomic data reflect protein abundance at the translational level but not post-translational modifications or enzymatic activities, and the identified candidate proteins (CRT, PDI, ACCase, PYR/PYL) remain correlative, as we could not conduct functional validation (e.g., overexpression, knockout, or pharmacological inhibition) due to limited biological material. Third, we did not measure germination percentage, embryo growth, or viability at each sampling time point; this was a consequence of destructive sampling and scarce material, a common constraint for endangered species. We therefore prioritized comprehensive proteomic and physiological profiling over germination assays, and our inference of dormancy release is based on observed hormonal and metabolic changes along with proteomic signatures consistent with known markers of dormancy alleviation, rather than on direct evidence of germination. Despite these limitations, our findings offer a robust resource for generating hypotheses about dormancy-release mechanisms in this endangered species. Future studies should integrate environmental sensors to capture the soil–plant–atmosphere continuum, employ complementary techniques (e.g., qRT-PCR, Western blotting, heterologous expression) to validate candidate protein functions once sufficient material becomes available, and include germination experiments to directly confirm dormancy break.

### 4.7. Comparison with Previous Studies on T. chinensis var. mairei Seeds

The present study complements and extends previous work on *T. chinensis* var. *mairei* seed dormancy. Zhou et al. established that seeds of this species exhibit morphophysiological dormancy and characterized the seasonal dynamics of endogenous hormones (ABA, GA_3_, and IAA) under field conditions [23]. Our hormone data are broadly consistent with their findings: ABA increased significantly during the early and late stratification periods, GA_3_ increased from 0 to 3 months, and IAA peaked at 6 months. However, whereas Zhou et al. focused on embryo growth and germination [23], our study provides the first proteomic characterization of the full stratification process, identifying 3047 DEPs and revealing the coordinated regulation of protein processing in the endoplasmic reticulum, fatty acid metabolism, carbohydrate metabolism, peroxisome pathways, and hormone signal transduction.

Chen et al. conducted a multi-omics study of germinating seeds, integrating transcriptome, proteome, spatial metabolome, and spatial lipidome data [22]. Their study identified abscisic acid and gibberellic acid homeostasis as closely associated with gene transcription and protein translation during germination. Our study differs in several important respects. First, we focused on the stratification phase, which precedes visible germination, whereas Chen et al. (2023) analyzed germinating seeds. Second, our sampling design included four time points across the 9-month stratification period, enabling a temporal analysis of proteomic changes, whereas Chen et al. compared dormant and germinating seeds [22]. Third, we identified specific candidate proteins (e.g., CRT, PDI, ACCase, PYR/PYL, and SRK2) that may regulate the transition from dormancy to germination, providing a resource for future functional studies. Together, these studies provide complementary views of the molecular events underlying dormancy and germination in this endangered species.

## 5. Conclusions

(1) The 9-month natural stratification promoted progressive increases in ABA and GA_3_, with IAA peaking at mid-stratification. These hormonal dynamics are consistent with active embryonic growth and cell division, indicating active embryonic growth and cell division. Storage reserves (soluble sugars, starch, fat) were continuously mobilized, while soluble protein and free amino acids exhibited opposing trends consistent with reciprocal conversion. POD activity increased then declined, whereas ACP activity decreased then increased, reflecting distinct stage-specific shifts in oxidative stress responses and energy metabolism during stratification. Collectively, stratification is accompanied by metabolic and respiratory activation and reserve mobilization, which are consistent with progressive dormancy alleviation and embryo development.

(2) Using TMT-labeled quantitative proteomics, a total of 3047 DEPs were identified (*p* < 0.05). GO enrichment analysis showed that more DEPs were detected in the late stage of stratification. KEGG pathway analysis showed that these DEPs were predominantly associated with fatty acid biosynthesis and degradation, carbohydrate metabolism, endoplasmic reticulum protein processing, peroxisome pathways, and plant hormone signal transduction. These pathways are known to be involved in seed dormancy and germination in other species, but their specific roles in *T. chinensis* var. *mairei* remain to be validated.

(3) During 0–3 months, acetyl-CoA carboxylase down-regulation was consistent with active fatty acid degradation, while CAT up-regulation and enhanced POD activity were consistent with stress responses. During 3–6 months, PYR/PYL and SRK2 up-regulation suggested activated ABA signaling and elevated ABA levels, along with increased glyceraldehyde-3-phosphate dehydrogenase expression indicating enhanced glycolysis and carbohydrate metabolism. During 6–9 months, calreticulin up-regulation and SRK2 down-regulation, together with the physiological and metabolic shifts observed, were consistent with dormancy alleviation, although functional validation of these candidate proteins remains to be performed.

## Data Availability

The original contributions presented in this study are included in the article. Further inquiries can be directed to the corresponding authors.
